# Higher Thyroid fT3-to-fT4 Ratio Is Associated with Gestational Diabetes Mellitus and Adverse Pregnancy Outcomes

**DOI:** 10.3390/jcm11175016

**Published:** 2022-08-26

**Authors:** Lore Raets, Caro Minschart, Annick Van den Bruel, Emmelien Van den Bogaert, Paul Van Crombrugge, Carolien Moyson, Johan Verhaeghe, Sofie Vandeginste, Hilde Verlaenen, Chris Vercammen, Toon Maes, Els Dufraimont, Nele Roggen, Christophe De Block, Yves Jacquemyn, Farah Mekahli, Katrien De Clippel, Anne Loccufier, Annouschka Laenen, Roland Devlieger, Chantal Mathieu, Brigitte Decallonne, Katrien Benhalima

**Affiliations:** 1 Department of Endocrinology, University Hospital Gasthuisberg, KU Leuven, Herestraat 49, 3000 Leuven, Belgium; 2 Department of Endocrinology, AZ St Jan Brugge, Ruddershove 10, 8000 Brugge, Belgium; 3 Department of Medicine, Katholieke Universiteit Leuven, Herestraat 49, 3000 Leuven, Belgium; 4 Department of Endocrinology, OLV-Ziekenhuis Aalst-Asse-Ninove, Moorselbaan 164, 9300 Aalst, Belgium; 5 Department of Obstetrics & Gynecology, UZ Gasthuisberg, Katholieke Universiteit Leuven, Herestraat 49, 3000 Leuven, Belgium; 6 Department of Obstetrics & Gynecology, OLV-Ziekenhuis Aalst-Asse-Ninove, Moorselbaan 164, 9300 Aalst, Belgium; 7 Department of Endocrinology, Imelda Ziekenhuis, Imeldalaan 9, 2820 Bonheiden, Belgium; 8 Department of Obstetrics & Gynecology, Imelda Ziekenhuis, Imeldalaan 9, 2820 Bonheiden, Belgium; 9 Department of Endocrinology-Diabetology-Metabolism, Antwerp University Hospital, Drie Eikenstraat 655, 2650 Edegem, Belgium; 10 Department of Obstetrics & Gynecology, Antwerp University Hospital (UZA), Drie Eikenstraat 655, 2650 Edegem, Belgium; 11 Antwerp Surgical Training, Anatomy and Research Centre, Global Health Institute GHI, Antwerp University (UA), 2000 Antwerp, Belgium; 12 Department of Endocrinology, Kliniek St-Jan Brussel, Kruidtuinlaan 32, 1000 Brussel, Belgium; 13 Department of Obstetrics & Gynecology, Kliniek St-Jan Brussel, Kruidtuinlaan 32, 1000 Brussel, Belgium; 14 Center of Biostatics and Statistical Bioinformatics, Katholieke Universiteit Leuven, Kapucijnenvoer 35 Bloc D, Box 7001, 3000 Leuven, Belgium

**Keywords:** gestational diabetes mellitus, thyroid function, fT3-to-fT4 ratio, free T3, pregnancy outcomes

## Abstract

Aim: To determine the association between thyroid function and the risk of developing gestational diabetes mellitus (GDM) and adverse pregnancy outcomes. Methods: This case–control study was a sub-analysis of the BEDIP-N study, in which 199 GDM women were matched for age and body mass index with 398 controls. Thyroid-stimulating hormone (TSH), free thyroxine (fT4), free triiodothyronine (fT3), and thyroid peroxidase (TPO) antibodies were measured at 6–14 weeks and 26–28 weeks during pregnancy. TSH and fT4 were also measured in early postpartum in GDM women. Results: The fT3-to-fT4 ratio at 26–28 weeks was positively associated with GDM risk with an adjusted odds ratio (aOR for smoking, education, parity, ethnicity, gestational weight gain, and (family) history of diabetes or GDM) of 2.12 (95% CI 1.07; 4.23), comparing the highest with the lowest tertile. Higher fT3 levels and a higher fT3-to-fT4 ratio were associated with a less favorable metabolic profile with higher BMI and more insulin resistance during pregnancy and postpartum. Women in the upper fT3 tertile and the upper fT3-to-fT4 ratio had a higher rate of preeclampsia [4.6% (10) vs. 1.0% (2), *p* = 0.040, and 4.4% (9) vs. 0.5% (1), *p* = 0.020], gestational hypertension [8.3% (18) vs. 3.1% (6), *p* = 0.034 and 8.9% (18) vs. 2.0% (4), *p* = 0.003], and caesarean sections [29.4% (63) vs. 16.1% (31), *p* = 0.002 and 32.2% (65) vs. 12.7% (25), *p* < 0.001]. Conclusion: A higher fT3-to-fT4 ratio late into pregnancy was associated with GDM, adverse pregnancy outcomes, and an adverse metabolic profile in early postpartum.

## 1. Introduction

Thyroid hormones (THs) are important for intrauterine fetal development, especially for the development of the fetal brain. During the early stages of pregnancy, the fetus largely depends on maternal THs [1]. Untreated overt maternal hypothyroidism during pregnancy leads to an increased risk of adverse pregnancy and child outcomes [2,3]. There has been a debate on the need for universal screening for thyroid dysfunction before or during pregnancy. The major negative impact of universal screening would be the identification of women with mild hypothyroidism, a population in whom the benefit of thyroid hormone supplementation is not well established [2]. 

THs play an important role in glucose metabolism. It has been suggested that thyroid dysfunction might be involved in the development of gestational diabetes mellitus (GDM) [4,5,6]. Sparse and conflicting data allude to the increased incidence of GDM in women with hypothyroidism [5,7,8,9]. Studies have shown that thyroid diseases in the first trimester are positively related to GDM [10,11]. Free T3 (fT3) levels may be an indicator of GDM risk starting early during pregnancy [5]. More data are needed to evaluate the association between thyroid function during pregnancy with GDM, adverse pregnancy outcomes, and risk of developing glucose intolerance in early postpartum. Moreover, there are currently only a few studies on the association between TH markers and GDM based on the 2013 WHO criteria [12,13]. We therefore aimed to determine the association between the fT3-to-fT4 ratio and related TH markers in early and late pregnancy and the risk of developing GDM, adverse pregnancy outcomes, and glucose intolerance in early postpartum. 

## 2. Materials and Methods

### 2.1. Study Design and Setting

This case–control study was a sub-analysis of the Belgian Diabetes in Pregnancy study (BEDIP-N) (NCT02036619). We selected all women with GDM and matched them with women with normal glucose tolerance (two controls for every GDM case). Women with a history of thyroid disease were excluded. The controls were normal glucose tolerant (NGT) women matched for age and body mass index (BMI) in early pregnancy. Age matching was performed using a maximum range of 2 years and ages > 40 were rounded to 40 years. BMI matching was performed using a maximum range of 2 points and BMI > 35 was rounded to 35. 

BEDIP-N was a multicentric prospective cohort study that has previously been described in detail [14,15,16,17,18]. The study was approved by the Institutional Review Boards of all participating centers, and all investigations were carried out in accordance with the principles of the Declaration of Helsinki. Informed consent was obtained before inclusion in the study. Participants were enrolled between 6 and 14 weeks of pregnancy, when fasting plasma glucose (FPG) was measured. Women with impaired fasting glycaemia (IFG) or diabetes in early pregnancy according to the American Diabetes Association (ADA)-criteria were excluded [19]. Women without (pre)diabetes received universal screening for GDM between 24 and 28 weeks of pregnancy with a non-fasting 50 g glucose challenge test (GCT) followed by a 75 g 2 h OGTT. The 2013 WHO criteria were used for the diagnosis of GDM [14,15,20]. The ADA-recommended glycemic targets were used for the treatment of GDM [20]. Women with GDM received an invitation for a postpartum 75 g OGTT 6 to 16 weeks after delivery. The ADA criteria were used to define diabetes and glucose intolerance postpartum [IFG and/or impaired glucose tolerance (IGT)] [14,20]. 

### 2.2. Study Visits and Measurements

Baseline characteristics and obstetrical history were collected at first visit [14]. At first visit and at the time of the OGTT, anthropometric measurements were obtained, and several self-administered questionnaires were completed [14]. 

A BMI ≥ 25 kg/m^2^ was defined as overweight, and a BMI ≥ 30 kg/m^2^ was defined as obese based on the BMI at first prenatal visit. A fasting blood test was taken to measure FPG, insulin, lipid profiles (total cholesterol, HDL and LDL cholesterol, triglycerides), and HbA1c. The homeostasis model assessment of insulin resistance (HOMA-IR) and beta-cell function (HOMA-B) was carried out [21]. The results of glucose and insulin levels during the OGTT were used to calculate the Matsuda index, which is a measure of whole body insulin sensitivity [14,21,22,23,24,25].

### 2.3. Pregnancy and Delivery Outcome Data

The following pregnancy outcomes were collected: gestational age at delivery, preeclampsia (de novo BP ≥ 140/90 mmHg > 20 weeks with proteinuria or signs of end-organ dysfunction), gestational hypertension (de novo BP ≥ 140/90 mmHg > 20 weeks), type of labor, type of delivery, macrosomia (>4 kg), large-for-gestational age (LGA) defined as birth weight > 90 percentile or small-for-gestational age (SGA) defined as birth weight < 10 percentile (according to standardized Flemish birth charts adjusted for sex of the baby and parity) [26], shoulder dystocia, preterm delivery (<37 completed weeks), and admission on the neonatal intensive care unit (NICU) [14]. A glycemic value < 2.2 mmol/L was considered as a neonatal hypoglycemia across all centers. The difference in weight between the first prenatal visit and the time of the OGTT was calculated as an early weight gain. Total gestational weight gain (GWG) was calculated as the difference in weight between first prenatal visit and delivery. The excessive total GWG (EGWG) was defined according to the 2009 Institute of Medicine (IOM) guidelines [27].

### 2.4. Analytical Methods

In line with routine clinical practice in each center, TSH was measured between 6 and 14 weeks of pregnancy. In women with GDM, TSH and fT4 were also measured at the postpartum visit by the local laboratory of each center [electrochemiluminescence immunoassay (ECLIA) method, Roche Cobas e801]. The following fasting samples were analyzed by the laboratory of UZ Leuven: between 6 and 14 weeks: fT4, fT3, and TPO levels; between 26 and 28 weeks: TSH, fT4, and fT3 levels; and in women with GDM, fT3 was also analyzed postpartum. Concentrations of serum TSH (mIU/L), fT3 (pmol/L), fT4 (ng/dL), and TPO antibodies (IU/mL) were measured using the ECLIA method on the Roche Cobas e801 analyzer with Roche reagents (Roche Diagnostics, Indianapolis, IN, USA). The fT3/fT4 ratio was obtained by dividing serum concentrations of fT3 by fT4. 

### 2.5. Statistical Analysis 

Descriptive statistics were presented as frequencies and percentages for categorical variables and means with standard deviations or medians with an interquartile range for continuous variables. Categorical variables were analyzed using the chi-square test or the Fisher exact test in the case of low (<5) cell frequencies, whereas continuous variables were analyzed using the Kruskal–Wallis test for data with a non-normal distribution or one-way ANOVA test for data with a normal distribution.

To estimate crude and adjusted odds ratios (aORs) of GDM for each thyroid marker accounting for the matched case–control pairs, conditional logistic regression was used. Thyroid markers were both analyzed continuously and in tertiles. The tertiles were based on the thyroid marker distributions among the controls. ORs were calculated separately for the visit early during pregnancy (6–14 weeks) and at the time of the OGTT (26–28 weeks). For the multivariable models, we selected covariates including demographic factors and risk factors for GDM: smoking, education, parity, ethnicity, GWG, first-degree family history of diabetes, and a history of GDM. A correction for confounders (for non-white origin, education, smoking, and a history of impaired glucose intolerance) for the pregnancy outcomes was only applied to caesarian section (CS) and EGWG, as only these outcomes had a sufficient numbers of events. As TPO antibodies may influence both THs as well as glucose homeostasis, we repeated the analysis with exclusion of TPO-antibody-positive cases. A *p*-value < 0.05 was considered significant. Analyses were performed by statistician A. Laenen using SAS software.

## 3. Results

**Figure 1:**
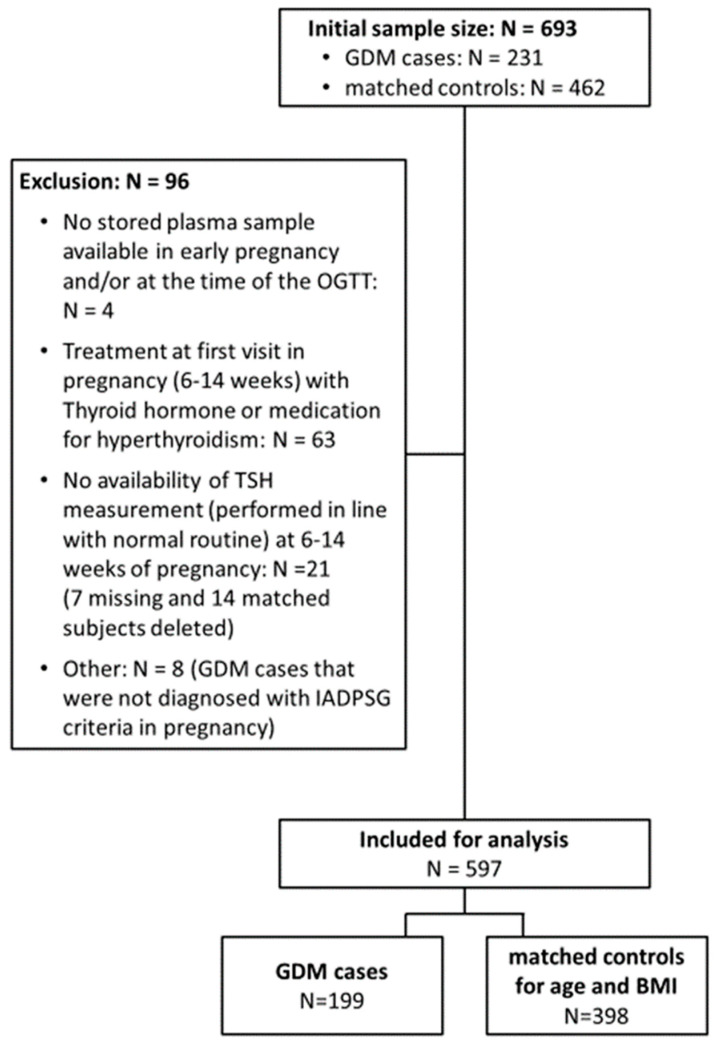
Flow chart of participants included in the sub-analysis. GDM: gestational diabetes mellitus; OGTT: oral glucose tolerance test; TSH: thyroid-stimulating hormone; IADPSG: International Association of the Diabetes and Pregnancy Study Groups; BMI: body mass index.

Two hundred and thirty-one women with GDM were matched, each to two controls based on age and BMI. Of this group, ninety-six (13.8%) were excluded for multiple reasons (Figure 1). In total, 597 women were included, of which 199 were GDM cases and 398 were matched controls. Compared to the NGT group, women with GDM smoked more before pregnancy, were more likely to have a first-degree family member with diabetes, had a more frequent history of GDM, and had a lower total weight gain (Table A1). TSH levels did not differ significantly between GDM cases and controls (Table 1). Mean fT4 levels were significantly lower among GDM women in the first trimester but not in the third trimester, whereas mean fT3 levels were significantly higher in the third but not in the first trimester (Table 1). Both fT3 and the fT3-to-fT4 ratio were positively associated with GDM in late pregnancy (Table 2). Women in the highest tertile of the fT3 levels had more frequent GDM compared with those in the lowest tertile. This association was no longer significant after adjusting for confounders, such as smoking, education, parity, ethnicity, GWG, first-degree family history of diabetes, and a history of GDM. The positive association between the fT3-to-fT4 ratio and GDM remained significant after adjustment with an aOR of 2.12 (95% CI 1.07; 4.23), comparing the highest with the lowest tertile. TSH levels were not associated with GDM (Table 2).

Increases in fT3 and the fT3-to-fT4 ratio were associated with a higher BMI, fasting glycaemia, post-load glycaemia, HOMA-IR, and fasting triglycerides and decreasing fasting HDL and ISSI-2 both in early and late pregnancy. Increasing fT3 was associated with decreasing fasting LDL in late pregnancy (Table 3).

Women in the upper fT3 tertile (52.9%) had a higher BMI, more often hypertension, higher HOMA-IR, lower HDL cholesterol, and higher fasting triglycerides at the first prenatal visit. At the OGTT, women in the upper tertile of fT3 more frequently had a diagnosis of GDM, EGWG, preeclampsia, gestational hypertension, a higher rate of cesarean sections (CS), and NICU admissions (Table 4). After adjusting for the previously mentioned confounders, only the rate of CS remained significantly higher in the upper tertile fT3 group [aOR 1.937, 95% CI (1.12; 3.36), *p* = 0.0187]. Women with GDM in the upper tertile fT3 group (61.6%) had a worse metabolic profile at the postpartum OGTT compared to women with GDM with lower fT3 (38.4%), but the rate of glucose intolerance was similar in both groups (Table A2). 

Compared to women in the lower fT3-to-fT4 tertile [49.2%] group, women in the upper fT3-to-fT4 tertile [50.7%] group had a higher BMI, a higher waist circumference, and a worse lipid profile in early pregnancy, and were more insulin-resistant (Table 5). These metabolic parameters remained worse at the time of the OGTT, and GDM was significantly more often diagnosed in the upper fT3-to-fT4 tertile group [38.9% (79) vs. 26.9% (53), *p* = 0.011] compared to the lower fT3-to-fT4 tertile group. EGWG, preeclampsia, gestational hypertension, CS, and LGA occurred more often in the upper fT3-to-fT4 tertile group compared to the lower fT3-to-fT4 tertile group (Table 5). After adjusting for confounders, the risk of developing CS and EGWG was no longer significantly higher. There were no significant differences in the rate of glucose intolerance postpartum between both groups (Table A3). 

## 4. Discussion

Existing data are conflicting on the association between GDM and THs during pregnancy. We found that both fT3 and the fT3-to-fT4 ratio in late pregnancy were significantly and positively associated with GDM. After adjusting for confounders such as smoking, education, parity, ethnicity, GWG, and a (first-degree family) history of diabetes or GDM, the association between the highest tertile of fT3 with GDM was no longer significant. In contrast, the fT3-to-fT4 ratio remained significantly associated with GDM after adjusting for confounders. FT4 early into pregnancy was negatively correlated with GDM after adjusting for confounders.

There is evidence that THs play an important role in glucose metabolism by regulating hepatic gluconeogenesis, in the intestinal absorption of glucose, and in the uptake of glucose in peripheral tissues. It is suggested that thyroid dysfunction may have a role in the emergence of GDM [4,5]. However, data are sparse on the association between different thyroid function markers and GDM based on the 2013 WHO criteria [12,13]. Data are also limited on the impact of thyroid function within the normal range on pregnancy outcomes and the risk of developing glucose intolerance in early postpartum. 

Our results do not support previous studies in which TSH, even within the normal range in the first trimester, was positively associated with GDM [10,11]. This discrepant finding could be explained by differences in the study population (e.g., Caucasian vs. Chinese women), the study design, diagnostic criteria for GDM, and the sample size. However, our results are in line with earlier findings which show a negative correlation between fT4 concentration in early pregnancy and GDM [12,28,29,30]. Maternal fT4 is important for fetal development in the first trimester of pregnancy. In normal pregnancy, human chorionic gonadotrophin (HCG) stimulates the thyroid gland, which leads to a small and transient increase in maternal fT4. Mothers with thyroid insufficiency might be more prone to pregnancy complications (e.g., GDM) compared to euthyroid women [28,31]. A study of Rawal et al. showed that higher fT3 levels may be an indicator of GDM risk starting early into pregnancy [5]. A recent Chinese study showed that both peripheral and central thyroid resistance indices during the first half of pregnancy significantly affected the subsequent risk of GDM [13]. This study showed that higher levels of fT3 and a higher fT3-to-fT4 ratio were associated with increased GDM risk, even among euthyroid women [13]. However, this study investigates a Chinese population, though we also present the first data on the impact of TH markers on the risk of developing GDM in a Caucasian population using the 2013 WHO criteria. 

Our study also provides information on the association between thyroid markers, insulin resistance, and beta-cell function during pregnancy and postpartum. In contrast to other studies [5,12,13], in our sub-analysis, the association between higher tertile fT3 was no longer significant after adjusting for confounders. Discrepancies between the results in our study and the Rawal study may be related to differences in the study population as a multiracial pregnancy cohort from the US was evaluated using the Carpenter and Coustan diagnostic criteria in the Rawal study, potentially resulting in the diagnosis of more severe degrees of hyperglycaemia compared to our study using the 2013 WHO criteria, identifying milder forms of GDM. Our study also had a substantially larger sample size. 

FT3 is the primary biologically active hormone involved in glucose homeostasis. The increase in the fT3 hormone during pregnancy is responsible for stimulating endogenous glucose production. Of all circulating fT3, 80% is formed by peripheral deiodinase (DIO) activity, which is indicated by an increase in the fT3-to-fT4 ratio both in a normal pregnancy as well as in GDM pregnancies [5,32]. Furthermore, studies on euthyroid patients without diabetes showed a positive relation between FPG and fT3 as well as between FPG and the fT3-to-fT4 ratio, indicating that THs may play an important role in the different pathophysiological mechanisms of IFG and IGT [33,34]. It could also be hypothesized that changes in placental DIO activity could contribute to an increase in the fT3-to-fT4 ratio, e.g., when there is a decrease in DIO3 as a result of (mal)adaptation of the placenta or an accelerated placental maturation in women with GDM [31]. In addition, an Italian study found a single-nucleotide polymorphism Thr921a in the coding region of *DIO2* gene to be associated with insulin resistance, lower glucose disposal, and increased BMI [35,36]. However, there is still controversy on the exact role of polymorphisms on the *DOI2* gene and its association with glucose tolerance [37,38]. Our observational study cannot demonstrate any causal relationship between THs and GDM, but rather suggests that THs may play a role in the pathophysiology of GDM, and its related adverse pregnancy outcomes and the risk of developing adverse metabolic profile postpartum. 

Women in the upper fT3 tertile and in the upper fT3-to-fT4 ratio group had a worse metabolic profile compared to women in the lower tertiles, both in early and later stages of pregnancy and for women with GDM also in early postpartum. Studies in NGT pregnant women without a history of thyroid dysfunction showed that a higher fT3-to-fT4 ratio was associated with a less favorable metabolic phenotype with a positive association between the fT3-to-fT4 ratio and BMI, triglycerides, and HOMA-IR, as also shown in our population [32,39]. Our results are also in line with findings in euthyroid middle-aged subjects, in which a higher fT3 and a higher fT3-to-fT4 ratio were associated with an unfavorable metabolic profile [34]. Additionally, our study demonstrated that women with a higher fT3 and higher fT3-to-fT4 ratio had more adverse pregnancy outcomes with higher rates of EGWG, preeclampsia, gestational hypertension, CS, LGA, and NICU admissions. After adjusting for confounders, the association between the upper tertile of the fT3-to-fT4 ratio and the risk of developing CS and EGWG and the association between the upper tertile fT3 and EGWG were no longer significant. Recent studies have shown a relationship between preeclampsia, gestational hypertension, CS, LGA, and NICU admissions in pregnant women with subclinical hypothyroidism [40,41,42,43,44]. Our study provides novel data showing that adverse pregnancy outcomes might also be related to THs within the normal range of fT3 and the fT3-to-fT4 ratio.

At the postpartum OGTT, women with GDM in the upper tertile fT3 and the upper fT3-to-fT4 ratio group had a persistently worse metabolic profile. However, the rate of glucose intolerance was similar between the lower and highest tertiles groups. To our knowledge, we present the first data on the metabolic profiles and the risk of developing glucose intolerance in early postpartum, according to different degrees of fT3 and the fT3-to-fT4 ratio during pregnancy. A study from China showed an association between thyroid antibodies (TPO-antibody and thyroglobulin antibody) and glucose metabolism postpartum, but no data on thyroid function postpartum were available [12]. 

A major strength of our study is the large multicentric prospective cohort with a large detailed dataset containing broad demographic, clinical, and obstetrical outcomes. We provide the first data on the association between thyroid markers and GDM diagnosed with the 2013 WHO criteria in euthyroid women in a Caucasian population. Additionally, GDM cases were matched for age and BMI with NGT controls. Data on the risk of developing GDM and adverse pregnancy outcomes were adjusted for important confounders. Women with hypothyroidism were excluded. Most TH markers were centrally analyzed by the same laboratory of UZ Leuven. A limitation of the study is that our cohort was mainly Caucasian, with a relatively low background risk of developing GDM. In addition, we had no data on β-HCG levels, iodine status, and neonatal thyroid function parameters. 

## 5. Conclusions

In conclusion, we found that a higher fT3-to-fT4 ratio late during pregnancy is associated with GDM, adverse pregnancy outcomes, and a persistent worse metabolic profile in early postpartum.

## Figures and Tables

**Table 1:** Mean plasma concentrations of thyroid function markers in women with GDM and matched controls.

	Total Cohort N = 597	GDM N = 199 (33.3%)	NGT N = 398 (66.7%)	*p*-Value
**6–14 weeks**				
Gestational age at blood collection (weeks)	11.9 ± 1.6	12.0 ± 1.7	11.9 ± 1.6	0.571
TSH (mIU/L)	1.3 ± 0.9	1.3 ± 0.9	1.3 ± 0.9	0.416
fT4 (pmol/L)	14.9 ± 2.4	14.6 ± 2.1	15.0 ±2.6	**0.039**
fT3 (pmol/L)	5.0 ± 0.8	5.0 ± 0.6	5.0 ± 0.8	0.288
Anti-TPO ≥ 34 IU/L (%)	6.5 (39)	6.6 (13)	6.5 (26)	1.000
fT3/fT4 (upper vs. lower tertile)	0.34 ± 0.06	0.35 ± 0.05	0.34 ± 0.06	**0.020**
**24–28 weeks**				
Gestational age at blood collection (weeks)	26.9 ± 1.1	27.1 ± 1.2	26.9 ± 1.0	**0.034**
TSH (mIU/L)	1.6 ± 0.8	1.5 ± 0.8	1.6 ± 0.8	0.104
fT4 (pmol/L)	12.2 ± 1.6	12.1 ± 1.6	12.3 ± 1.6	0.146
fT3 (pmol/L)	4.4 ± 0.5	4.5 ± 0.5	4.4 ± 0.5	**0.013**
fT3/fT4 (upper vs. lower tertile)	0.36 ± 0.06	0.37 ± 0.07	0.36 ± 0.06	**0.010**
**Change from 6–14 weeks to 26–28 weeks**				
TSH (mIU/L)	0.3 ± 0.8	0.3 ± 0.8	0.3 ± 0.8	0.394
fT4 (pmol/L)	−2.6 ± 1.9	−2.5 ± 1.5	−2.7 ± 2.0	0.396
fT3 (pmol/L)	−0.7 ± 0.6	−0.6 ± 0.5	−0.7 ± 0.7	0.075
fT3/fT4 (upper vs. lower tertile)	0.02 ± 0.1	0.02 ± 0.0	0.02 ± 0.1	0.336

GDM: gestational diabetes mellitus; NGT: normal glucose tolerant; TSH: thyroid-stimulating hormone; fT3: free triiodothyronine 3; fT4: free thyroxine 4; TPO: thyroid peroxidase. Categorical variables are presented as frequencies % (n); continuous variables are presented as mean ± SD. *p*-values < 0.05 are considered significant. Bold *p*-values are significant *p* < 0.05.

**Table 2:** Adjusted odds ratio’s for GDM according to tertiles of fT3, fT4, the fT3/fT4 ratio, and TSH.

	GDM N = 199 (33.3%)	NGT N = 398 (66.7%)	Crude Model	Multivariable Model ^a^
**6–14 weeks**				
fT3 (pmol/L)				
Tertile 1: 3.3–4.6	28.6 (57)	29.1 (116)	1	1
Tertile 2:4.7–5.1	30.6 (61)	32.7 (130)	0.96 (0.61; 1.49)	1.36 (0.73; 2.55)
Tertile 3: 5.2–13.8	40.7 (81)	38.2 (152)	1.09 (0.71; 1.69)	0.75 (0.38; 1.46)
Upper decile: 5.8–13.8	28.7 (23)	25.2 (39)	0.76 (0.32; 1.78)	0.99 (0.16; 6.28)
*p* for trend			0.977	0.249
Per unit increment			1.00 (0.80; 1.26)	0.77 (0.50; 1.18)
fT4 (pmol/L)				
Tertile 1: 9.9–13.7	35.2 (70)	29.9 (119)	1	1
Tertile 2: 13.8–15.3	32.7 (65)	32.7 (130)	0.84 (0.56; 1.27)	0.56 (0.31; 1.01)
Tertile 3: 15.4–47.1	32.2 (64)	37.4 (149)	0.72 (0.47; 1.10)	0.57 (0.32; 1.02)
Upper decile: 17.2–47.1	21.3 (19)	27.4 (45)	0.72 (0.32; 1.61)	0.54 (0.17; 1.77)
*p* for trend			0.078	0.111
Per unit increment			0.93 (0.85; 1.01)	0.91 (0.81; 1.02)
fT3/fT4 ratio				
Tertile 1: 0.20–0.31	30.1 (60)	34.4 (137)	1	1
Tertile 2: 0.32–0.35	31.2 (62)	34.2 (136)	1.07 (0.70; 1.65)	1.29 (0.68; 2.42)
Tertile 3: 0.36–1.01	38.7 (77)	31.4 (125)	1.50 (0.95; 2.36)	1.31 (0.69; 2.48)
Upper decile: 0.41–1.01	29.4 (25)	20.3 (35)	1.26 (0.45; 3.52)	0.74 (0.14; 3.80)
*p* for trend			0.070	0.467
Per unit increment			20.03 (0.079; 510.43)	5.00 (0.06; 383.24)
TSH (mIU/L)				
Tertile 1: 0.00–0.85	36.7 (73)	31.6 (124)	1	1
Tertile 2: 0.86–1.59	33.2 (66)	37.2 (146)	0.79 (0.52; 1.20)	1.07 (0.59; 1.98)
Tertile 3: 1.60–8.00	30.1 (60)	31.1 (122)	0.80 (0.52; 1.23)	1.15 (0.63; 2.10)
Upper decile: 2.40–8.00	18.0 (16)	17.3 (26)	0.84 (0.34; 2.06)	1.79 (0.37; 8.74)
*p* for trend			0.576	0.651
Per unit increment			0.95 (0.78; 1.15)	1.06 (0.81; 1.39)
**24–28 weeks**				
fT3 (pmol/L)				
Tertile 1: 3.0–4.1	26.6 (53)	34.9 (139)	1	1
Tertile 2: 4.2–4.5	30.6 (61)	32.2 (128)	1.29 (0.83; 2.01)	1.63 (0.89; 2.98)
Tertile 3: 4.6–7.2	42.7 (85)	32.9 (131)	**1.74 (1.13; 2.67)**	1.70 (0.91; 3.16)
Upper decile: 5.0–7.2	39.8 (35)	22.3 (40)	1.52 (0.71; 3.24)	0.82 (0.17; 3.92)
*p* for trend			**0.014**	0.174
Per unit increment			**1.55 (1.09; 2.21)**	1.43 (0.85; 2.38)
fT4 (pmol/L)				
Tertile 1: 8.0–11.4	33.2 (66)	30.9 (123)	1	1
Tertile 2: 11.5–12.7	36.7 (73)	32.7 (130)	1.06 (0.70; 1.61)	0.84 (0.44; 1.57)
Tertile 3: 12.8–24.0	30.1 (60)	36.4 (145)	0.78 (0.51; 1.18)	0.77 (0.43; 1.39)
Upper decile: 14.3–24.0	18.5 (15)	26.8 (45)	0.65 (0.24; 1.80)	1.30 (0.28; 6.00)
*p* for trend			0.157	0.467
Per unit increment			0.92 (0.83; 1.03)	0.94 (0.80; 1.11)
fT3/fT4 ratio				
Tertile 1: 0.21–0.33	26.6 (53)	36.2 (144)	1	1
Tertile 2: 0.34–0.37	33.7 (67)	32.7 (130)	1.45 (0.94; 2.24)	1.82 (0.98; 3.40)
Tertile 3: 0.38–0.66	39.7 (79)	31.2 (124)	**1.85 (1.18; 2.92)**	**2.12 (1.07; 4.23)**
Upper decile: 0.44–0.66	37.6 (32)	16.8 (29)	1.92 (0.67; 5.52)	2.12 (0.34; 13.00)
*p* for trend			**0.001**	**0.042**
Per unit increment			**190.42 (8.09; 4483.4)**	**134.84 (1.19; 15302)**
TSH (mIU/L)				
Tertile 1: 0.04–1.20	38.2 (76)	30.0 (119)	1	1
Tertile 2: 1.21–1.84	29.1 (58)	34.5 (137)	0.64 (0.41; 0.98)	0.54 (0.29; 1.04)
Tertile 3: 1.85–5.95	32.7 (65)	35.5 (141)	0.69 (0.45; 1.06)	0.89 (0.49; 1.63)
Upper decile: 2.60–5.95	20.0 (19)	25.6 (41)	0.76 (0.31; 1.87)	1.56 (0.38; 6.45)
*p* for trend			0.145	0.713
Per unit increment			0.84 (0.66; 1.06)	1.06 (0.77; 1.47)

GDM: gestational diabetes mellitus; NGT: normal glucose tolerant; TSH: thyroid-stimulating hormone; fT3: free triiodothyronine; fT4: free thyroxine. Upper decile: ≥90th percentile. Boldface indicates statistically significant results. ^a^ Adjusted for smoking, education, parity, ethnicity, gestational weight gain, first-degree family history of diabetes, and history of GDM.

**Table 3:** Bivariate correlations between maternal thyroid function tests at 26–28 weeks of pregnancy and selected parameters in women with GDM and their matched controls.

	fT3		fT3/fT4 Ratio	
	r	*p*	r	*p*
**General**				
Age (years)	−0.076	0.063	0.021	0.610
**6–14 weeks**				
BMI (kg/m^2^)	0.327	**<0.001**	0.365	**<0.001**
Waist circumference (cm)	0.269	**<0.001**	0.320	**<0.001**
Systolic blood pressure (mmHg)	0.278	**<0.001**	0.230	**<0.001**
Diastolic blood pressure (mmHg)	0.225	**<0.001**	0.195	**<0.001**
Fasting glycaemia (mmol/L)	0.131	**0.001**	0.162	**<0.001**
Fasting insulin (pmol/L)	0.249	**<0.001**	0.294	**<0.001**
HOMA-IR	0.257	**<0.001**	0.305	**<0.001**
HOMA-B	0.071	0.085	0.143	**<0.001**
HbA1c (mmol/mol and %)	0.022	0.597	0.078	0.057
Fasting Total cholesterol (mmol/L)	−0.045	0.276	0.112	**0.006**
Fasting HDL (mmol/L)	−0.196	**<0.001**	−0.156	**<0.001**
Fasting LDL (mmol/L)	0.011	0.793	0.135	**0.001**
Fasting TG (mmol/L)	0.126	**0.002**	0.252	**<0.001**
**26–28 weeks**				
BMI (kg/m^2^)	0.345	**<0.001**	0.400	**<0.001**
Systolic blood pressure (mmHg)	0.300	**<0.001**	0.318	**<0.001**
Diastolic blood pressure (mmHg)	0.273	**<0.001**	0.265	**<0.001**
Glucose non-fasting 0 min on GCT (mmol/L)	0.069	0.095	0.122	**0.003**
Glucose 60 min on GCT (mmol/L)	0.070	0.091	0.092	**0.025**
Fasting glycaemia OGTT(mmol/L)	0.173	**<0.001**	0.242	**<0.001**
30 min glucose OGTT (mmol/L)	0.136	**0.001**	0.105	**0.010**
1 h glucose OGTT (mmol/L)	0.120	**0.003**	0.171	**<0.001**
2 h glucose OGTT (mmol/L)	0.093	**0.023**	0.134	**0.001**
Fasting insulin OGTT (pmol/L)	0.285	**<0.001**	0.379	**<0.001**
30 min insulin OGTT (pmol/L)	0.176	**<0.001**	0.200	**<0.001**
1 h insulin OGTT (pmol/L)	0.207	**<0.001**	0.319	**<0.001**
2 h insulin OGTT (pmol/L)	0.205	**<0.001**	0.297	**<0.001**
HbA1c (mmol/mol and %)	0.142	**<0.001**	0.260	**<0.001**
Matsuda insulin sensitivity	−0.269	**<0.001**	−0.349	**<0.001**
HOMA-IR	0.290	**<0.001**	0.395	**<0.001**
HOMA-B	0.106	**0.009**	0.124	**0.002**
ISSI-2	−0.194	**<0.001**	−0.259	**<0.001**
Insulinogenic index/HOMA-IR	−0.179	**<0.001**	−0.226	**<0.001**
Fasting total cholesterol (mmol/L)	−0.190	**<0.001**	−0.038	0.356
Fasting HDL (mmol/L)	−0.213	**<0.001**	−0.180	**<0.001**
Fasting LDL (mmol/L)	−0.136	**0.001**	−0.037	0.369
Fasting TG (mmol/L)	0.074	0.071	0.206	**<0.001**
**Delivery**				
Total weight gain (delivery—first visit) (kg)	−0.021	0.629	0.021	0.622
Weight baby (g)	0.014	0.734	0.008	0.838

GDM: gestational diabetes mellitus; BMI: body mass index; OGTT: oral glucose tolerance test; HOMA-IR: homeostasis model assessment of insulin resistance; HOMA-B: homeostasis model assessment for beta cell function; ISSI-2: insulin secretion sensitivity index-2; HDL: high-density lipoprotein; LDL: low-density lipoprotein; TG: triglycerides; overweight: BMI ≥ 25–29.9 kg/m^2^; obesity: BMI ≥ 30 kg/m^2^. Differences are considered significant at *p*-value < 0.05. Bold *p*-values are significant *p* < 0.05.

**Table 4:** Distribution of selected patient characteristics according to lower and upper tertiles of fT3.

	fT3 Lower Tertile (3.0–4.1) 26–28 Weeks N = 192 (47.1%)	fT3 Upper Tertile (4.6–7.2) 26–28 Weeks N = 216 (52.9%)	*p*-Value
**General**			
Age (years)	32.1 ± 4.0	31.2 ± 4.7	**0.047**
**6–14 weeks visit**			
BMI (kg/m^2^)	23.9 ± 4.4	27.7 ± 6.0	**<0.001**
% Overweight	30.2 (58)	61.6 (133)	**<0.001**
% Obesity	8.3 (16)	32.9 (71)	**<0.001**
Waist circumference (cm)	84.1 ± 11.0	91.9 ± 13.6	**<0.001**
% Waist 80–88 cm	44.9 (84)	26.5 (56)	**<0.001**
% Waist ≥ 88 cm	22.5 (42)	53.5 (113)	
Weight gain (first visit till OGTT) (kg)	6.7 ± 3.3	6.7 ± 3.4	0.525
Systolic blood pressure (mmHg)	112.3 ± 8.7	119.4 ± 12.1	**<0.001**
Diastolic blood pressure (mmHg)	69.2 ± 7.1	73.5 ± 8.9	**<0.001**
% Hypertension (≥140/90 mmHg)	0.5 (1)	6.9 (15)	**<0.001**
Fasting glycaemia (mmol/L)	2.15 (2.02–2.22)	2.17 (2.07–2.27)	**0.004**
Fasting insulin (pmol/L)	41.1 (31.0–53.8)	60.1 (43.0–86.8)	**<0.001**
HOMA-IR	8.2 (6.3–11.3)	12.2 (8.6–18.5)	**<0.001**
HOMA-B	760.7 (582.1–1052.9)	1015.4 (735.8–1519.6)	**<0.001**
HbA1c (mmol/mol and %)	31.1 (30.1–33.3) 5.0 (4.9–5.2)	31.1 (30.1–33.3) 5.0 (4.9–5.2)	0.779
Fasting total cholesterol (mmol/L)	2.2 (2.0–2.4)	2.1 (1.9–2.4)	0.400
Fasting HDL (mmol/L)	0.85 (0.75–0.96)	0.77 (0.68–0.88)	**<0.001**
Fasting LDL (mmol/L)	1.15 (0.91–1.32)	1.38 (0.94–1.33)	0.655
Fasting TG (mmol/L)	0.96 (0.79–1.20)	1.04 (0.86–1.45)	**0.002**
fT4 (pmol/L)	14.7 (13.4–16.1)	14.8 (13.5–16.0)	0.477
TSH (mIU/L)	1.2 (0.8–1.8)	1.1 (0.6–1.8)	0.136
% Anti-TPO-positive (≥34 IU/L)	6.8 (13)	6.0 (13)	0.840
Total score lifestyle			
Physical activity	1.0 (0–2.0)	1.0 (0–2.0)	0.073
Diet	3.0 (0–5.0)	2.0 (0–4.0)	**0.009**
**24–28 weeks visit**			
BMI (kg/m^2^)	26.3 ± 4.4	30.2 ± 5.7	**<0.001**
% Overweight	51.6 (95)	78.4 (167)	**<0.001**
% Obesity	16.3 (30)	47.9 (102)	**<0.001**
Systolic blood pressure (mmHg)	109.5 ± 8.7	116.0 ± 11.4	**<0.001**
Diastolic blood pressure (mmHg)	64.9 ± 6.5	69.6 ± 8.4	**<0.001**
% Hypertension (≥140/90 mmHg)	0.5 (1)	4.6 (10)	**0.012**
Weeks of gestation at OGTT	26.9 ± 1.1	27.0 ± 1.1	0.685
Fasting glycaemia OGTT (mmol/L)	4.38 (4.16–4.66)	4.50 (4.30–4.82)	**<0.001**
30 min glucose OGTT (mmol/L)	7.21 (6.49–8.10)	7.55 (6.72–8.49)	**0.003**
1 h glucose OGTT (mmol/L)	7.52 (6.19–9.02)	8.27 (6.94–9.43)	**0.007**
2 h glucose OGTT (mmol/L)	6.60 (5.44–7.85)	7.16 (5.88–8.27)	**0.008**
% GDM diagnosis	27.6 (53)	39.3 (85)	**0.016**
Fasting insulin OGTT (pmol/L)	56.4 (41.2–78.2)	79.9 (61.3–113.7)	**<0.001**
30 min insulin OGTT (pmol/L)	442.4 (336.4–677.50)	583.1 (407.7–872.1)	**<0.001**
1 h insulin OGTT (pmol/L)	542.8 (382.3–828.8)	693.7 (478.8–1014.0)	**<0.001**
2 h insulin OGTT (pmol/L)	513.9 (344.3–786.0)	692.0 (452.8–1005.0)	**<0.001**
HbA1c (mmol/mol and %)	30.1 (29.0–32.2) 4.9 (4.8–5.1)	31.1 (30.1–33.3) 5.0 (4.9–5.2)	**0.002**
Matsuda insulin sensitivity	0.6 (0.4–0.8)	0.4 (0.3–0.5)	**<0.001**
HOMA-IR	11.0 (7.9–15.9)	16.1 (11.7–23.5)	**<0.001**
HOMA-B	1281.4 (913.4–1759.6)	1635.1 (1147.3–2292.2)	**<0.001**
ISSI-2	0.1 (0.1–0.3)	0.1 (0–0.2)	**<0.001**
Insulinogenic index/HOMA-IR	0.3 (0.2–0.5)	0.3 (0.2–0.3)	**<0.001**
Fasting total cholesterol (mmol/L)	6.47 (5.74–7.15)	5.97 (5.46–6.70)	**<0.001**
Fasting HDL (mmol/L)	2.03 (1.75–2.31)	1.82 (1.59–2.08)	**<0.001**
Fasting LDL (mmol/L)	3.59 (2.93–4.29)	3.21 (2.75–3.90)	**0.004**
Fasting TG (mmol/L)	1.84 (1.46–2.25)	1.95 (1.63–2.48)	**0.015**
GDM treatment *			
GDM	27.6 (53)	39.3 (85)	**0.016**
% Need for treatment with insulin	3.7 (7)	4.6 (10)	1.000
Gestational age at start insulin	30.7 ± 3.0	29.6 ± 2.0	0.551
**Delivery**			
% Excessive weight gain	21.3 (38)	36.2 (64)	**0.002**
Gestational age (weeks)	39.3 ± 1.6	38.9 ± 1.9	**0.036**
% Preeclampsia	1.0 (2)	4.6 (10)	**0.040**
% Gestational hypertension	3.1 (6)	8.3 (18)	**0.034**
% Cesarean sections (total)	16.1 (31)	29.4 (63)	**0.002**
% Planned CS	4.7 (9)	16.8 (36)	**<0.001**
% Emergency CS (during labor)	11.5 (2)	12.6 (27)	0.762
% Macrosomia (>4 kg)	6.2 (12)	8.4 (18)	0.451
% LGA	8.8 (17)	13.9 (30)	0.122
% SGA	5.7 (11)	4.6 (10)	0.659
% Neonatal hypoglycaemia <2.22 mmol/L	5.8 (10)	9.8 (18)	0.172
% NICU admission	6.8 (13)	14.9 (32)	**0.011**

GDM: gestational diabetes mellitus; BMI: body mass index; OGTT: oral glucose tolerance test; HOMA-IR: homeostasis model assessment of insulin resistance; HOMA-B: homeostasis model assessment for beta cell function; HDL: high-density lipoprotein; LDL: low-density lipoprotein; TG: triglycerides; LGA: large-for-gestational age infant; SGA: small-for-gestational age infant; NICU: neonatal intensive care unit; overweight: BMI ≥ 25–29.9 kg/m^2^; obesity: BMI ≥ 30 kg/m^2^. * Data only available for women with GDM. Categorical variables are presented as frequencies %(n); continuous variables are presented as mean ± SD if normally distributed and as median ± IQR if not normally distributed; differences are considered significant at *p*-value < 0.05. Bold *p*-values are significant *p* < 0.05.

**Table 5:** Distribution of patient characteristics according to lower and upper tertiles of the fT3-to-fT4 ratio.

	fT3-to-fT4 Ratio Lower Tertile (0.21 to 0.33) 26–28 Weeks N = 197 (49.2%)	fT3-to-fT4 Ratio Upper Tertile (0.38 to 0.66) 26–28 Weeks N = 203 (50.7%)	*p*-Value
**General**			
Age (years)	31.6 ± 4.1	31.8 ± 4.6	0.752
% History of impaired glucose intolerance	0.6 (1)	4.9 (9)	**0.020**
**6–14 weeks visit**			
Weeks of gestation first visit with FPG	12.0 ± 1.6	11.9 ± 1.7	0.669
BMI (kg/m^2^)	23.3 ± 3.9	28.0 ± 5.6	**<0.001**
% Overweight	27.4 (54)	66.0 (134)	**<0.001**
% Obesity	6.1 (12)	32.5 (66)	
Waist circumference (cm)	83.4 ± 10.0	92.6 ± 12.5	**<0.001**
% Waist 80–88 cm	42.0 (81)	30.6 (61)	**<0.001**
% Waist ≥ 88 cm	22.3 (43)	55.8 (111)	
Weight gain (first visit till OGTT) (kg)	6.4 ± 3.3	7.2 ± 3.0	**0.007**
Systolic blood pressure (mmHg)	112.4 ± 9.0	118.2 ± 11.5	**<0.001**
Diastolic blood pressure (mmHg)	69.3 ± 7.3	72.7 ± 9.0	**<0.001**
% Hypertension (≥140/90 mmHg)	0.5 (1)	6.9 (14)	**<0.001**
Fasting glycaemia (mmol/L)	4.61 (4.33–4.77)	4.66 (4.50–4.88)	**0.002**
Fasting insulin (pmol/L)	39.4 (30.6–53.6)	62.5 (43.9–89.1)	**<0.001**
HOMA-IR	8.0 (6.1–11.2)	12.9 (9.0–18.7)	**<0.001**
HOMA-B	742.9 (582.6–1052.5)	1048.9 (774.0–1470.9)	**<0.001**
HbA1c (mmol/mol and %)	31.1 (30.1–33.3) 5.0 (4.9–5.2)	31.1 (30.1–33.3) 5.0 (4.9–5.2)	0.053
Fasting total cholesterol (mmol/L)	4.54 (3.98–5.11)	4.75 (4.31–5.26)	**0.006**
Fasting HDL (mmol/L)	1.82 (1.57–2.03)	1.67 (1.44–1.90)	**<0.001**
Fasting LDL (mmol/L)	2.31 (1.85–2.70)	2.49 (2.11–2.93)	**<0.001**
Fasting TG (mmol/L)	0.91 (0.77–1.13)	1.10 (0.86–1.57)	**<0.001**
fT4 (pmol/L)	15.7 (14.4–16.9)	13.8 (12.8–14.9)	**<0.001**
TSH (mIU/L)	1.1 (0.60; 1.75)	1.2 (0.8–1.9)	**0.039**
% Anti-TPO-positive (≥34 IU/L)	6.6 (13)	7.9 (16)	0.702
**24–28 weeks visit**			
BMI (kg/m^2^)	25.6 ± 3.9	30.7 ± 5.3	**<0.001**
% Overweight	44.8 (7)	86.4 (171)	**<0.001**
% Obesity	12.9 (25)	50.0 (99)	**<0.001**
Systolic blood pressure (mmHg)	109.7 ± 9.0	116.6 ± 11.2	**<0.001**
Diastolic blood pressure (mmHg)	65.2 ± 6.9	69.7 ± 8.2	**<0.001**
% Hypertension (≥140/90 mmHg)	0 (0)	4.5 (9)	**0.004**
Fasting glycaemia OGTT(mmol/L)	4.38 (4.16–4.61)	4.55 (4.33–4.88)	**<0.001**
30 min glucose OGTT (mmol/L)	7.21 (6.44–7.99)	7.46 (6.83–8.38)	**0.006**
1 h glucose OGTT (mmol/L)	7.49 (6.05–8.82)	8.32 (6.99–9.49)	**<0.001**
2 h glucose OGTT (mmol/L)	6.60 (5.38–7.88)	7.16 (5.94–8.27)	**0.004**
Fasting insulin OGTT (pmol/L)	53.7 (39.8–76.7)	84.8 (64.3–124.9)	**<0.001**
30 min insulin OGTT (pmol/L)	466.9 (325.5–661.2)	591.9 (418.9–867.9)	**<0.001**
1 h insulin OGTT (pmol/L)	518.9 (357.2–741.0)	752.4 (563.2–1038.0)	**<0.001**
2 h insulin OGTT (pmol/L)	497.8 (334.7–737.6)	757.0 (499.2–1046.5)	**<0.001**
% GDM diagnosis	26.9 (53)	38.9 (79)	**0.011**
HbA1c (mmol/mol and %)	30.1 (29.0–32.2) 4.9 (4.8–5.1)	31.1 (30.1–33.3) 5.0 (4.9–5.2)	**<0.001**
Matsuda insulin sensitivity	0.6 (0.4–0.8)	0.4 (0.3–0.5)	**<0.001**
HOMA-IR	10.7 (7.7–15.4)	17.5 (12.5–25.8)	**<0.001**
HOMA-B	1254.5 (889.6–1699.7)	1668.0 (1146.0–2311.7)	**<0.001**
ISSI-2	0.2 (0.1–0.3)	0.1 (0.0–0.1)	**<0.001**
Insulinogenic index/HOMA-IR	0.3 (0.2–0.5)	0.2 (0.2–0.3)	**<0.001**
Fasting total cholesterol (mmol/L)	6.09 (5.60–6.60)	6.14 (5.62–6.91)	0.937
Fasting HDL (mmol/L)	1.98 (1.72–2.29)	1.77 (1.51–2.11)	**<0.001**
Fasting LDL (mmol/L)	3.36 (2.80–3.93)	3.31 (2.80–3.98)	0.961
Fasting TG (mmol/L)	1.73 (1.39–2.16)	2.04 (1.69–2.56)	**<0.001**
Increase (difference) in TG between the first and second visit (mmol/L)	0.76 (0.50–1.08)	0.87 (0.59–1.24)	**0.033**
fT4 (pmol/L)	13.3 (12.5–14.3)	11.1 (10.4–11.8)	**<0.001**
TSH (mIU/L)	1.4 (1.0–2.0)	1.7 (1.1–2.1)	**0.005**
Total score lifestyle:			
Physical activity	1.0 (0–2.0)	1.0 (0–2.0)	0.216
Diet	3.0 (1.0–5.0)	2.0 (0–4.0)	**0.004**
GDM treatment *			
GDM	26.9 (53)	38.9 (79)	**0.011**
% Need for treatment with insulin	3.0 (6)	5.9 (12)	0.540
Gestational age at start insulin	29.8 ± 1.2	30.2 ± 1.6	0.666
**Delivery**			
Total weight gain (delivery—first visit) (kg)	10.7 ± 4.9	11.6 ± 5.7	**0.038**
% Excessive weight gain	15.3 (27)	39.5 (70)	**<0.001**
Gestational age (weeks)	39.3 ± 1.5	39.1 ± 1.8	0.094
% Preeclampsia	0.5 (1)	4.4 (9)	**0.020**
% Gestational hypertension	2.0 (4)	8.9 (18)	**0.003**
% Preterm delivery	6.1 (12)	7.4 (15)	0.692
% Induction labor	27.9 (55)	37.1 (75)	0.055
% Cesarean sections (total)	12.7 (25)	32.2 (65)	**<0.001**
% Planned CS	6.1 (12)	17.3 (35)	**<0.001**
% Emergency CS (during labor)	6.6 (13)	14.8 (30)	**0.009**
% Macrosomia (>4 kg)	6.1 (12)	9.9 (20)	0.197
% LGA	8.1 (16)	17.8 (36)	**0.005**
% SGA	4.1 (8)	3.0 (6)	0.597
% Neonatal hypoglycaemia <2.22 mmol/L	5.6 (10)	8.7 (15)	0.304
% NICU admission	6.1 (12)	16.8 (34)	**<0.001**

GDM: gestational diabetes mellitus; BMI: body mass index; OGTT: oral glucose tolerance test; HOMA-B: homeostasis model assessment for beta cell function; HDL: high-density lipoprotein; LDL: low-density lipoprotein; TG: triglycerides; fT4: free T4; TSH: thyroid-stimulating hormone; LGA: large-for-gestational age infant; SGA: small-for-gestational age infant; NICU: neonatal intensive care unit; IFG: impaired fasting glycaemia; IGT: impaired glucose tolerance; overweight: BMI ≥ 25–29.9 kg/m^2^; obesity: BMI ≥ 30 kg/m^2^. * Data only available for women with GDM. Categorical variables are presented as frequencies % (n); continuous variables are presented as mean ±SD if normally distributed and as median ± IQR if not normally distributed; odds ratios with 95% confidence intervals are presented for significant differences; differences are considered significant at *p*-value < 0.05. Bold *p*-values are significant *p* < 0.05.

## Data Availability

Original data generated and analyzed during this study are included in this published article.

## Appendix A

**Table A1:** Distribution of patient characteristics among women who had GDM and their matched controls.

	NGT N = 398 (66.7%)	GDM N = 199 (33.3%)	*p*-Value
**General**			
Age (years)	31.3 ± 4.3	31.9 ± 4.7	0.174
% Ethnic minorities			0.432
Asian	3.8 (15)	6.1 (12)	
Northern African	6.0 (24)	4.1 (8)	
Turkish	1.5 (6)	1.5 (3)	
Black African	1.3 (5)	2.0 (4)	
Middle East	1.5 (6)	0.5 (1)	
Latin American	2.0 (8)	0.5 (1)	
Other	2.5 (10)	4.1 (8)	
% Multiparity	49.5 (194)	52.8 (105)	0.488
% Higher degree diploma	82.0 (300)	76.0 (136)	0.111
% Highest education:			0.220
primary school	0.8 (3)	3.2 (6)	
till 15 years	4.9 (19)	4.7 (9)	
high school	18.0 (70)	20.5 (39)	
bachelor	36.0 (140)	36.3 (69)	
master	40.4 (157)	35.3 (67)	
% Paid job	87.4 (346)	91.3 (178)	0.170
% Smoking before pregnancy	26.5 (105)	35.5 (70)	**0.028**
% Smoking during pregnancy	2.5 (10)	5.6 (11)	0.096
% First-degree family history of diabetes	11.4 (43)	18.6 (34)	**0.026**
% First-degree family history of GDM	4.4 (16)	7.6 (14)	0.163
% History of GDM *	5.1 (10)	29.8 (31)	**<0.001**
% History of impaired glucose intolerance	2.0 (7)	2.7 (5)	0.551
**6–14 weeks visit**			
Weeks of gestation first visit with FPG	11.9 ± 1.6	11.9 ± 1.7	0.571
BMI (kg/m^2^)	25.7 ± 5.4	26.4 ± 5.3	0.057
% Overweight	45.5 (181)	51.0 (101)	0.223
% Obesity	19.6 (78)	22.2 (44)	0.453
Weight gain (first visit till OGTT) (kg)	6.8 ± 3.2	6.9 ± 3.7	0.642
**Delivery**			
Total weight gain (delivery—first visit) (kg)	12.5 ± 5.2	8.7 ± 5.0	**<0.001**
% Excessive weight gain	35.1 (124)	15.8 (26)	**<0.001**

GDM: gestational diabetes mellitus; BMI: body mass index; OGTT: oral glucose tolerance test; overweight: BMI ≥ 25–29.9 kg/m^2^; obesity: BMI ≥ 30 kg/m^2^. * Data only available for women with GDM. Variables are presented as frequencies % (n); continuous variables are presented as mean ± SD if normally distributed and as median ± IQR if not normally distributed; differences are considered significant at *p*-value < 0.05. Bold *p*-values are significant at *p* < 0.05.

**Table A2:** Postpartum characteristics in women with GDM according to lower and upper tertiles of fT3.

	fT3 Lower Tertile (3.0–4.1) 26–28 Weeks N = 53 (38.4%)	fT3 Upper Tertile (4.6–7.2) 26–28 Weeks N = 85 (61.6%)	*p*-Value
**Postpartum**			
Glucose intolerance at postpartum OGTT			1.000
% IFG	4.2 (2)	5.6 (4)	
% IGT	12.5 (6)	11.1 (8)	
% Both IFG and IGT	0 (0)	1.4 (1)	
BMI (kg/m^2^) *	23.6 ± 4.0	28.8 ± 5.6	**<0.001**
% Overweight *	27.1 (13)	73.9 (51)	**<0.001**
% Obesity *	6.2 (3)	34.8 (24)	**<0.001**
Systolic blood pressure (mmHg) *	112.3 ± 10.1	120.0 ± 12.4	**0.002**
Diastolic blood pressure (mmHg) *	70.5 ± 9.1	75.6 ± 8.9	**0.006**
% Hypertension (≥140/90 mmHg)	2.1 (1)	8.7 (6)	0.237
Fasting glycaemia (mmol/L) *	4.72 (4.47–4.91)	4.94 (4.69–5.16)	**0.003**
30 min glucose OGTT (mmol/L) *	7.80 (6.94–8.74)	7.66 (6.77–8.60)	0.637
1 h glucose OGTT (mmol/L) *	7.30 (5.99–8.63)	7.08 (5.80–8.21)	0.488
2 h glucose OGTT (mmol/L) *	5.50 (4.94–6.66)	6.10 (5.00–6.91)	0.423
Fasting insulin (pmol/L) *	38.5 (23.6–58.8)	54.3 (39.5–84.2)	**0.001**
30 min insulin OGTT (pmol/L) *	332.2 (228.0–418.0)	388.2 (258.0–571.5)	0.104
1 h insulin OGTT (pmol/L) *	364.5 (273.3–501.4)	451.7 (269.2–694.8)	0.081
2 h insulin OGTT (pmol/L) *	249.0 (164.4–424.7)	338.4 (228.6–546.2)	**0.028**
HbA1c (%) *	5.3 (5.1–5.5)	5.2 (5.1–5.5)	0.820
Matsuda insulin sensitivity *	0.9 (0.5–1.4)	0.7 (0.4–0.9)	**0.006**
Stumvoll index *	−135.6 (−346.9–220.9)	95.2 (−164.6–534.1)	**0.017**
Oral disposition index *	−90.0 (−338.5–159.0)	69.0 (−136.3–212.3)	**0.020**
HOMA-IR *	8.1 (4.6–12.6)	11.2 (8.9–19.2)	**<0.001**
HOMA-B *	642.9 (447.7–826.5)	784.0 (582.4–1210.8)	**0.033**
ISSI-2 *	0.3 (0.2–0.7)	0.2 (0.1–0.4)	0.013
Insulinogenic index/Homa-IR *	0.3 (0.2–0.4)	0.2 (0.2–0.3)	**0.043**
Fasting total cholesterol (mmol/L) *	4.80 (4.13–5.21)	4.60 (4.11–5.08)	0.421
Fasting HDL (mmol/L) *	1.67 (1.44–1.95)	4.41 (1.26–1.67)	**0.003**
Fasting LDL (mmol/L) *	2.64 (2.16–3.13)	2.67 (2.13–3.13)	0.967
Fasting TG (mmol/L) *	0.75 (0.62–0.96)	0.89 (0.69–1.25)	**0.008**

GDM: gestational diabetes mellitus; BMI: body mass index; OGTT: oral glucose tolerance test; HOMA-IR: homeostasis model assessment of insulin resistance; HOMA-B: homeostasis model assessment for beta cell function; ISSI-2: insulin secretion-sensitivity index-2; HDL: high-density lipoprotein; LDL: low-density lipoprotein; TG: triglycerides; LGA: large-for-gestational age infant; SGA: small-for-gestational age infant; NICU: neonatal intensive care unit; IFG: impaired fasting glycaemia; IGT: impaired glucose tolerance; overweight: BMI ≥ 25–29.9 kg/m^2^; obesity: BMI ≥ 30 kg/m^2^. * Data only available for women with GDM. Categorical variables are presented as frequencies % (n); continuous variables are presented as mean ± SD if normally distributed and as median ± IQR if not normally distributed; differences are considered significant at *p*-value < 0.05. Bold *p*-values are significant at *p* < 0.05.

**Table A3:** Postpartum characteristics in GDM according to lower and upper tertiles of the fT3-to-fT4 ratio.

	GDM and fT3-to-fT4 Ratio Lower Tertile (0.21 to 0.33) 26–28 Weeks N = 53 (40.1%)	GDM and fT3-to-fT4 Ratio Upper Tertile (0.38 to 0.66) 26–28 Weeks N = 79 (59.8%)	*p*-Value
**Postpartum**			
Glucose intolerance at postpartum OGTT	17.8 (8)	19.1 (13)	0.721
% IFG	6.7 (3)	2.9 (2)	
% IGT	8.9 (4)	13.2 (9)	
% Both IFG and IGT	2.2 (1)	2.9 (2)	
BMI (kg/m^2^) *	23.1 ± 3.8	28.4 ± 5.6	**<0.001**
% Overweight *	22.2 (10)	68.7 (46)	**<0.001**
% Obesity *	6.7 (3)	31.3 (21)	**0.002**
Systolic blood pressure (mmHg) *	111.1 ± 14.0	120.6 ± 13.6	**<0.001**
Diastolic blood pressure (mmHg) *	70.6 ± 9.0	75.5 ± 8.5	**0.004**
% Hypertension (≥140/90 mmHg)	6.7 (3)	7.5 (5)	1.000
Fasting glycaemia (mmol/L) *	4.72 (4.50–4.94)	4.94 (4.74–5.19)	0.011
30 min glucose OGTT (mmol/L) *	7.66 (6.55–8.94)	7.83 (6.83–8.60)	0.887
1 h glucose OGTT (mmol/L) *	6.94 (6.10–8.60)	7.35 (5.72–8.38)	0.808
2 h glucose OGTT (mmol/L) *	5.55 (4.99–6.27)	5.91 (4.99–7.13)	0.480
Fasting insulin (pmol/L) *	38.8 (26.3–56.1)	55.4 (38.1–101.0)	**<0.001**
30 min insulin OGTT (pmol/L) *	332.2 (230.7–454.7)	418.5 (289.3–565.5)	0.055
1 h insulin OGTT (pmol/L) *	364.6 (229.2–554.1)	487.1 (324.5–790.0)	**0.036**
2 h insulin OGTT (pmol/L) *	280.4 (189.9–453.6)	342.8 (233.9–653.5)	**0.034**
HbA1c (%) *	5.2 (5.1–5.5)	5.3 (5.0–5.5)	0.590
Matsuda insulin sensitivity *	0.8 (0.5–1.3)	0.6 (0.4–0.9)	**0.002**
Stumvoll index *	−55.1 (−328.2–311.1)	185.8 (−30.8–660.7)	**0.016**
Oral disposition index *	−81.9 (−349.3–190.3)	97.6 (−35.8–254.9)	**0.018**
HOMA-IR *	8.3 (5.8–12.2)	12.1 (8.4–22.6)	**<0.001**
HOMA-B *	675.0 (445.9–1074.5)	787.7 (563.7–1287.3)	**0.050**
ISSI-2 *	0.3 (0.2–0.7)	0.2 (0.1–0.4)	**0.004**
Insulinogenic index/Homa-IR *	0.3 (0.2–0.4)	0.2 (0.1–0.3)	**0.047**
Fasting total cholesterol (mmol/L) *	4.87 (4.13–5.2)	4.74 (4.34–5.16)	0.869
Fasting HDL (mmol/L) *	1.63 (1.30–1.95)	1.39 (1.18–1.77)	**0.009**
Fasting LDL (mmol/L) *	2.66 (2.12–3.20)	2.81 (2.31–3.13)	0.516
Fasting TG (mmol/L) *	0.80 (0.64–0.97)	0.94 (0.64–1.43)	0.062
fT4 (pmol/L)	16.2 (14.7–17.9)	15.5 (14.1–16.6)	**0.040**
TSH (mIU/L)	1.5 (0.9–1.9)	1.5 (1.1–2.1)	0.733
Lifestyle score: physical activity *	1.0 (0–2.0)	1.0 (0–2.0)	0.879
Lifestyle score: diet *	3.0 (0–6.0)	2.0 (0–4.0)	0.293
% breastfeeding *	81.4 (35)	80.6 (54)	1.000
METs category at time of the OGTT *			0.161
% Low	19.5 (8)	12.3 (8)	
% Moderate	53.6 (22)	43.1 (28)	
% High	26.8 (11)	44.6 (29)	
% MET category low *	19.5 (8)	12.3 (8)	0.405

GDM: gestational diabetes mellitus; BMI: body mass index; OGTT: oral glucose tolerance test; HOMA-B: homeostasis model assessment for beta cell function; ISSI-2: insulin secretion-sensitivity index-2; HDL: high-density lipoprotein; LDL: low-density lipoprotein; TG: triglycerides; fT4: free T4; TSH: thyroid-stimulating hormone; MET: metabolic equivalent of task; LGA: large-for-gestational age infant; SGA: small-for-gestational age infant; NICU: neonatal intensive care unit; IFG: impaired fasting glycaemia; IGT: impaired glucose tolerance; overweight: BMI ≥ 25–29.9 kg/m^2^; obesity: BMI ≥ 30 kg/m^2^. * Data only available for women with GDM. Categorical variables are presented as frequencies % (n); continuous variables are presented as mean ± SD if normally distributed and as median ± IQR if not normally distributed; odds ratios with 95% confidence intervals are presented for significant differences; differences are considered significant at *p*-value < 0.05. Bold *p*-values are significant at *p* < 0.05.
